# Utilizing gentamicin concentrations to estimate glomerular filtration rate in intensive care unit patients

**DOI:** 10.1038/s41598-025-01256-z

**Published:** 2025-05-18

**Authors:** Anna-Karin Smekal, Maria Swartling, Elisabet I. Nielsen, Mia Furebring, Anders O. Larsson, Miklos Lipcsey

**Affiliations:** 1 Department of Surgical Sciences, Anaesthesiology and Intensive Care, Uppsala University, Sweden, USA; 2 Clinical Microbiology L2:02, Karolinska University Hospital, Solna, Stockholm, 171 76 Sweden; 3 Department of Pharmacy, Uppsala University, Uppsala, Sweden; 4 Department of Medical Sciences, Infectious Diseases, Uppsala University, Uppsala, Sweden; 5 Department of Medical Sciences, Clinical Chemistry, Uppsala University, Uppsala, Sweden; 6 Department of Surgical Sciences, Hedenstierna laboratory, Uppsala University, Uppsala, Sweden

**Keywords:** Gentamicin, Intensive care unit, Glomerular filtration rate, Biomarkers, Medical research, Nephrology

## Abstract

Estimated glomerular filtration rate (eGFR) based on creatinine (eGFR_Creatinine_) or cystatin C (eGFR_CystatinC_) require steady-state conditions and thus have limitations in intensive care unit (ICU) patients. Gentamicin is a potential exogenous marker for eGFR but poorly investigated. This retrospective study included adult ICU patients (*≥* 18 years) treated with gentamicin and not on renal replacement therapy (RRT) at admission. eGFR_Creatinine_ and eGFR_CystatinC_ were calculated using the LM-rev and CAPA equations, respectively. Gentamicin clearance was estimated using a population pharmacokinetic model and used as eGFR_Gentamicin_. Agreement between eGFRs vs. eGFR_Gentamicin_ and prediction of RRT and mortality for each eGFR were assessed. 254 patients were included of whom 11% (*n* = 28) received RRT later and 19% (*n* = 49) were dead at 30 days. The bias was 12 mL/min/1.73 m^2^ and 8 mL/min/1.73 m^2^, respectively, and the limits of agreement − 31–55 mL/min/1.73m^2^ and − 46–62 mL/min/1.73m^2^ for the agreement between eGFR_Gentamicin_ vs. eGFR_Creatinine_, and for eGFR_Gentamicin_ vs. eGFR_CystatinC_, respectively. The c-indexes for predicting RRT during ICU stay were 0.75 (0.64–0.86), 0.77 (0.66–0.88) and 0.80 (0.69–0.90) for eGFR_Creatinine_, eGFR_CystatinC_ and eGFR_Gentamicin_ respectively, and for 30-day mortality 0.61 (0.52–0.70), 0.61 (0.52–0.70) and 0.63 (0.54–0.72) respectively. In ICU patients already receiving gentamicin, eGFR_Gentamicin_ derived from population PK models can be used to assess renal function and could potentially help improve dosing of other renally cleared drugs like the β-lactams during early phase of infections in the ICU.

**Supplementary Information:**

The online version contains supplementary material available at 10.1038/s41598-025-01256-z.

## Introduction

A reliable assessment of the renal function in intensive care unit (ICU) patients with infections such as sepsis/septic shock, is important for several aspects of patient management. Without correct renal function measures, dosing to achieve adequate target organ levels of drugs with primarily renal elimination, such as many antimicrobial agents, is challenging. In critically ill patients, renal function varies both between and within patients since they are at risk of both augmented renal clearance (ARC) and acute kidney injury (AKI). ARC, a state of hyperfunctioning kidneys, presents a high risk of therapeutic failures for drugs with renal elimination such as the β-lactam antibiotics in the initial and most acute phase of infections in the ICU^1–4^. Pooled prevalence of ARC in the critically ill population has been reported to be 39% in one meta-analysis^4^. On the other hand, decreased renal function due to AKI can be seen in 60% of the patients with sepsis, and is associated with increased mortality risk and morbidity^5–7^, as well as risk for overdosing and toxicity of renally eliminated drugs.

The glomerular filtration rate (GFR) is regarded as the best indicator of global kidney function. GFR cannot be measured directly, instead, GFR can be assessed based on measured clearance (CL) of exogenous filtration markers as inulin, EDTA or iohexol, which are considered to be the reference methods^8^. However, these methods are time-consuming, expensive and impractical in unstable ICU patients. Instead, estimated GFR (eGFR) from serum levels of endogenous filtration markers such as creatinine (eGFR_Creatinine_) and cystatin C (eGFR_CystatinC_) are most commonly used in the ICU. Yet, these markers of renal function have limitations, particularly in critically ill patients without steady-state conditions and can both under and overestimate GFR^9–11^. The search for a better exogenous marker has been ongoing for years and clearance of aminoglycosides has been proposed as a possible option in critically ill patients already receiving this drug in the ICU because of their stable characteristics described below^9^.

Gentamicin is an aminoglycoside antibiotic that has been in use for parenteral administration since 1971^12^. Currently it is mostly used in combination therapy in cases of septic shock and Gram-negative infections to spare carbapenems. As gentamicin is freely filtered in the glomerulus, has little non-renal CL and is neither secreted nor reabsorbed in the kidney, it has potential as an exogenous marker of eGFR in patients already receiving this treatment^9^. Earlier studies evaluating if the elimination capacity of gentamicin is predictive of GFR have shown conflicting results^13–15^. The performance of estimated gentamicin clearance (eGFR_Gentamicin_) in predicting hard endpoints, associated with renal function, such as subsequent renal replacement therapy (RRT) during ICU stay and mortality has not been investigated previously.

Consequently, the aim of the study was to investigate how eGFR_Gentamicin_ derived from gentamicin serum concentrations by a population pharmacokinetic model corresponds to estimated eGFR using cystatin C (eGFR_CystatinC_) or creatinine (eGFR_Creatinine_) equations in an ICU setting. Moreover, to link these methods to renal function, we investigated the association between the three methods of eGFR and RRT initiated during ICU stay. Finally, we investigated the association between the three methods of eGFR and short (30-day) and long-term mortality since biomarkers of renal function are strong predictors of these^16^.

## Material and method

### Study design, patients and clinical data

This study was a retrospective observational study conducted between January 1st, 2009 and December 31st, 2013 in the general ICU of Uppsala University Hospital. Adult patients (≥ 18 years) given at least one gentamicin dose during their ICU stay with a corresponding sample of gentamicin concentration taken according to local guidelines were included. Exclusion criteria were age < 18 years or RRT before ICU stay. Demographic data as well as daily fluid balance, time on RRT during ICU stay, dose and administration time for gentamicin as well as time for gentamicin serum concentration sampling, were collected from the medical records. The corresponding results of plasma creatinine, cystatin C, and gentamicin concentrations following the first dose for each patient were collected from the laboratory information system of the Department of Clinical Chemistry and Pharmacology at Uppsala University Hospital. A follow-up was performed on July 8th, 2020, and the date of death (used for 30-day mortality) and overall mortality was collected from the patients’ medical records.

## Results

### Patients

In total 254 patients with a median age of 65.6 (IQR 54.3–74.2) years were included in the analysis and given one gentamicin dose (range 1–5). The 30-day mortality was 19% (*n* = 49) and 11% (*n* = 28) received RRT during the ICU stay. The patients’ clinical characteristics are presented in Table 1.

**Table 1:** Demographic and clinical characteristics of the included patients.

Characteristic	All patients (n = 254)
Age, year	65.6 (54.3–74.2)
Male gender	159 (63%)
Body weight, kg	82.0 (72.0–95.0)
BSA, m^2^	1.97 (1.82–2.10)
Gentamicin dose, mg/kg	3.2 (2.2-4.0)
Serum gentamicin concentration, mg/L	3.1 (2.0-4.4)
eGFR_Creatinine_, ml/min/1.73m^2^ BSA	55 (33–83)
eGFR_CystatinC_, ml/min/1.73m^2^ BSA	45 (28–71)
eGFR_Gentamicin_, ml/min/1.73m^2^ BSA	43 (27–62)
Renal placement therapy during ICU care	28 (11%)
30-day mortality	49 (19%)
Days from gentamicin sampling to death	1475 (63-3129)

BSA, body surface area; eGFR, estimated glomerular filtration rate; ICU, intensive care unit.

Data are presented as median (IQR) or number (percentages).

The individual CL was estimated with a median relative standard error of 21% (IQR 19–28%). eGFR values were similar in those with available height and weight data and those with missing data as well as in female and male patients (See Supplementary Table S1 and S2 online).

**Fig. 1:**
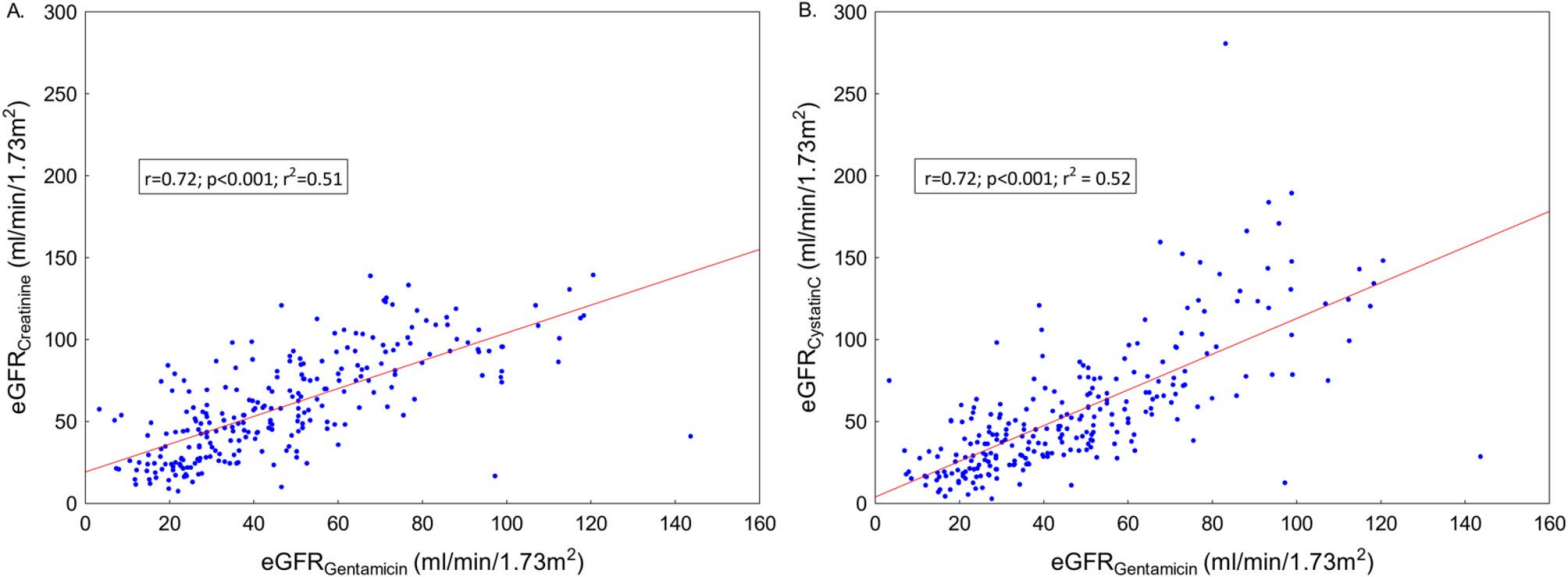
Scatter plot of the correlation between **A**) eGFR_Gentamicin_ and eGFR_Creatinine_ **B**) eGFR_Gentamicin_ and eGFR_CystatinC_. *eGFR; estimated glomerular filtration rate*.

The correlations between eGFR_Gentamicin_ and eGFR_Creatinine_ respectively eGFR_CystatinC_ are presented in Fig. 1. The correlation coefficients and coefficients of determination were similar for both analyses and indicated a positive linear relationship.

**Fig. 2:**
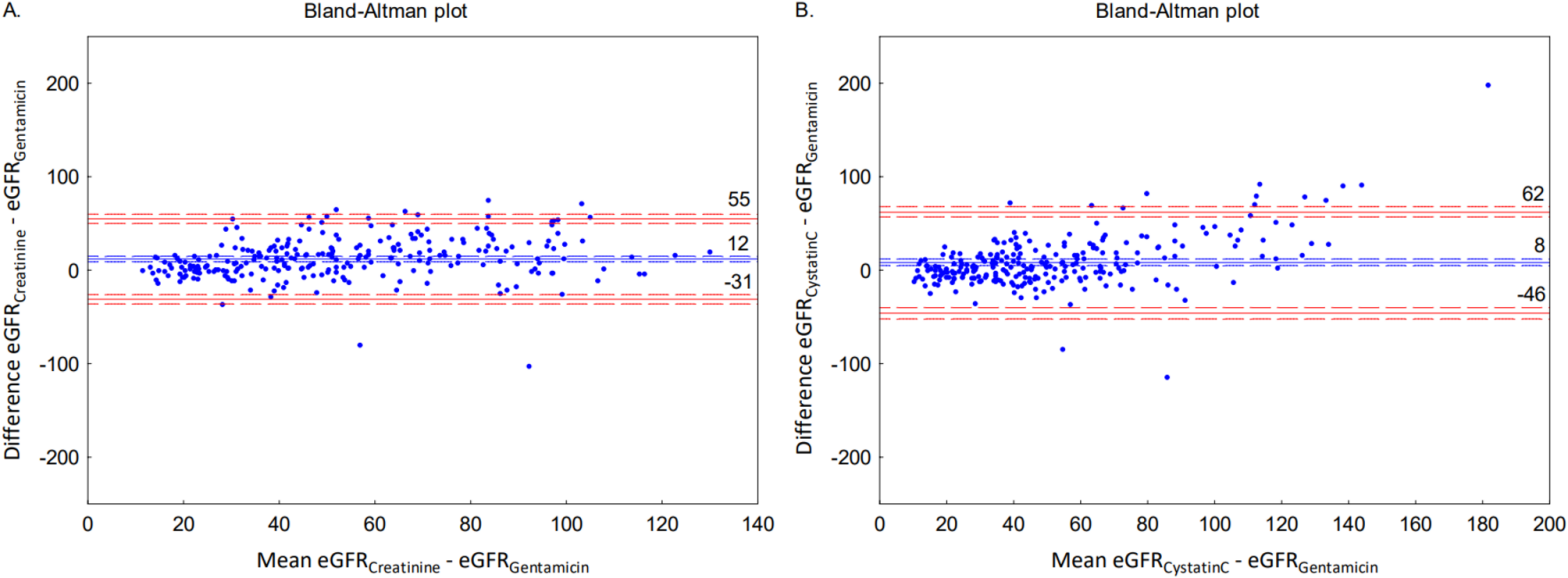
Bland-Altman plot of the agreement between **A**) eGFR_Gentamicin_ and eGFR_Creatinine_ **B**) eGFR_Gentamicin_ and eGFR_CystatinC_. The blue line marks the bias. Red lines mark the LoA. Dashed red respectively blue lines mark the 95% CI of the bias and the LoA. *eGFR; estimated glomerular filtration rate*,* LoA; limits of agreement*.

The agreement between eGFR_Gentamicin_ and eGFR_Creatinine_ respectively eGFR_CystatinC_ is presented as two Bland-Altman plots in Fig. 2.

For the agreement between eGFR_Gentamicin_ and eGFR_Creatinine_, the bias was 12 mL/min/1.73 m^2^ and the limits of agreement (LoA) 55 − 31 mL/min/1.73 m^2^. The corresponding results for the agreement between eGFR_Gentamicin_ and eGFR_CystatinC_ were 8 mL/min/1.73 m^2^ (bias) and 62 − 46 (LoA) mL/min/1.73 m^2^. The correlations between bias for eGFR_Creatinine_ vs. eGFR_Gentamicin_ and eGFR_CystatinC_ vs. eGFR_Gentamicin_ and weight were *r*=-0.37, r^2^ = 0.13 and *r*=-0.34, r^2^ = 0.11, respectively and the corresponding correlations for age were *r*=-0.05, r^2^ = 0.00 and *r*=-0.15, r^2^ = 0.02.

**Fig. 3:**
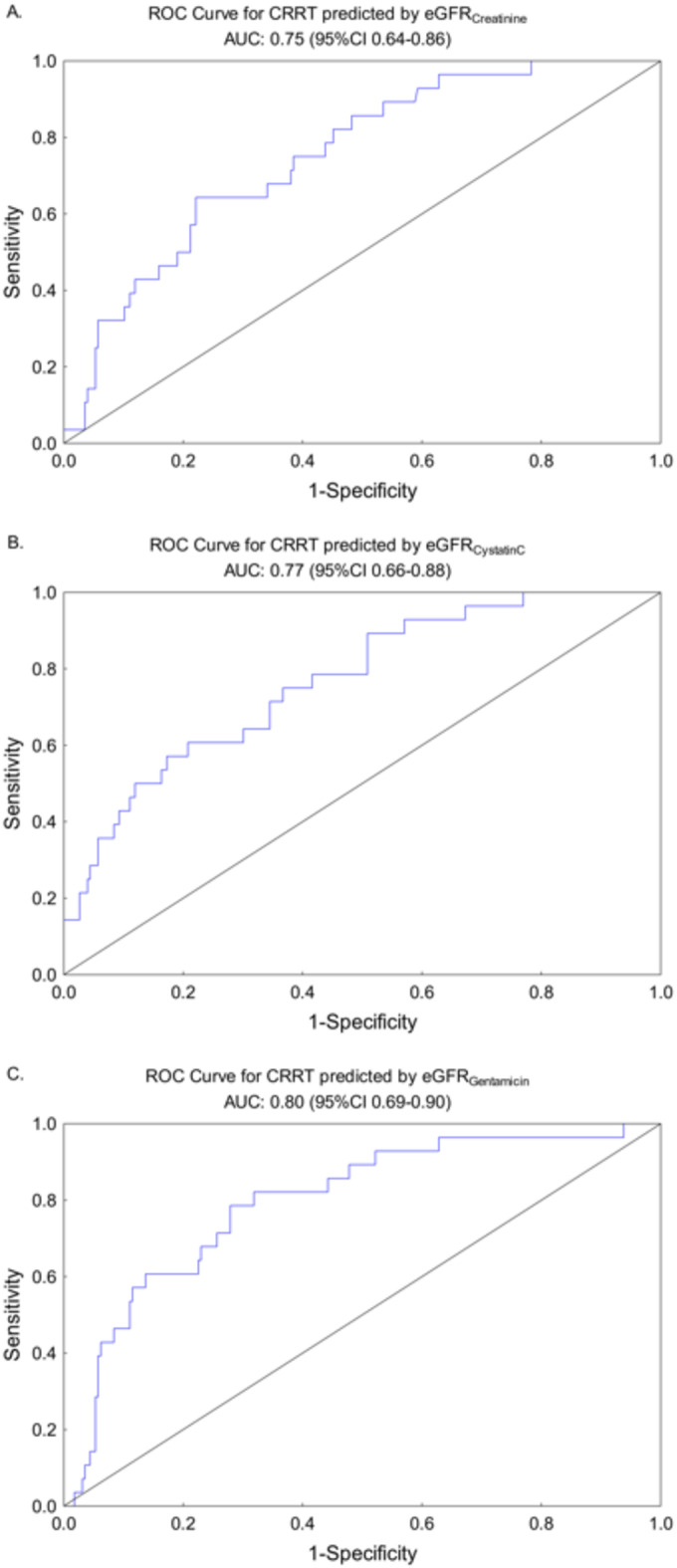
ROC-curve for RRT predicted by **A**) eGFR_Creatinine_ **B**) eGFR_CystatinC_ C) eGFR_Gentamicin_. *ROC; receiver operating characteristic*,* eGFR; estimated glomerular filtration rate*.

In Fig. 3, the performance of the three different eGFR methods to predict RRT during the ICU stay are presented as ROC curves.

The ROC-AUC or the c-index was 0.75 (0.64–0.86) for eGFR_Creatinine_, 0.77 (0.66–0.88) for eGFR_CystatinC_ and 0.80 (0.69–0.90) for eGFR_Gentamicin_. The corresponding odds ratios (OR) were 0.96 (0.94–0.98), 0.96 (0.93–0.98) and 0.94 (0.92–0.97).

**Fig. 4:**
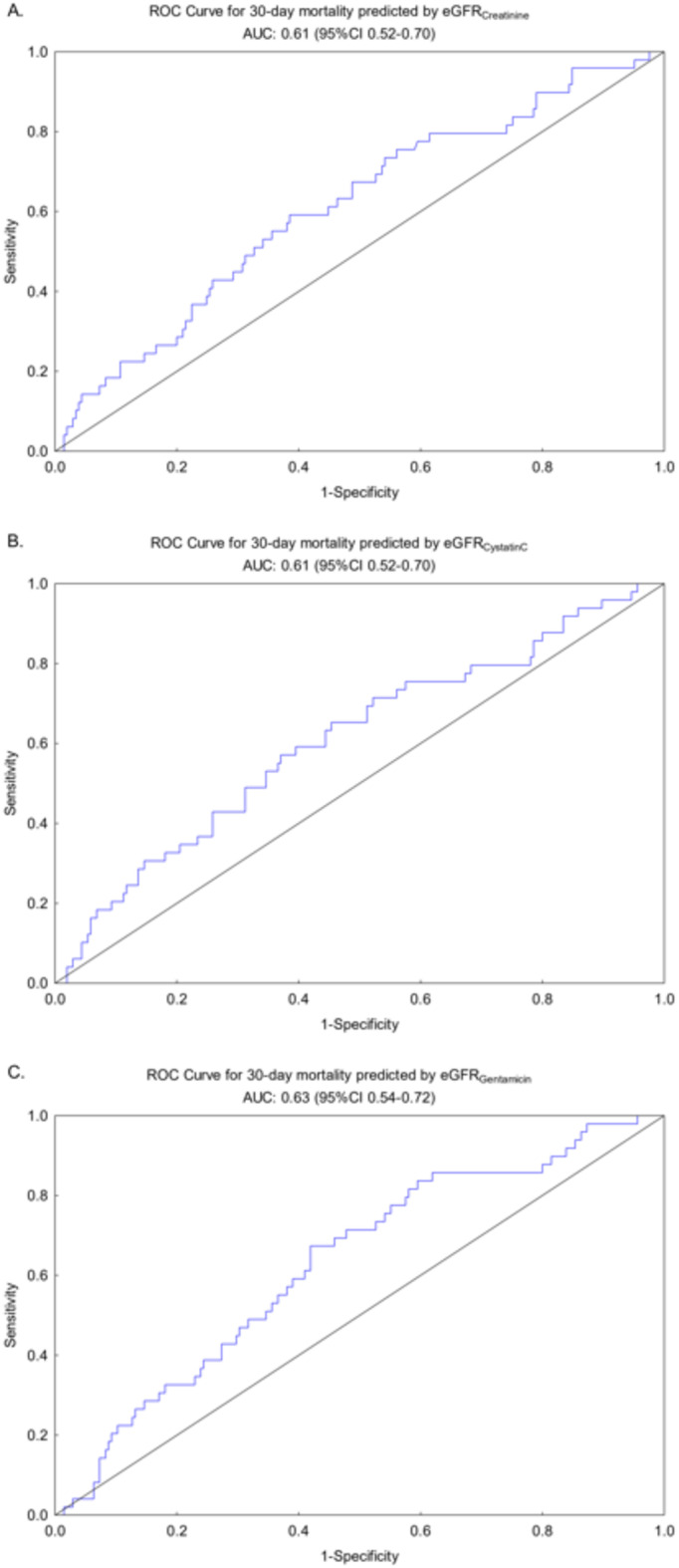
ROC-curve for 30-day mortality predicted by (A) eGFR_Creatinine_, (B) eGFR_CystatinC_, (C) eGFR_Gentamicin_ *ROC; receiver operating characteristic*,* eGFR; estimated glomerular filtration rate*.

The risk of death within 30 days after first given gentamicin dose in the ICU predicted by eGFR_Creatinine_, eGFR_CystatinC_ or eGFR_Gentamicin_ are presented as ROC curves in Fig. 4.

The c-indexes were 0.61 (0.52–0.70), 0.61 (0.52–0.70) and 0.63 (0.54–0.72) respectively. The OR were 0.99 (0.98-1.00), 0.99 (0.98-1.00) respectively 0.98 (0.97-1.00). The risk of death over time during the follow-up period calculated as hazard ratios (HR) were 0.99 (0.99-1.00) for eGFR_Creatinine_, 0.99 (0.99-1.00) for eGFR_CystatinC_ and 0.99 (0.98–0.99) for eGFR_Gentamicin_.

## Discussion

In a Swedish ICU cohort of 254 patients, treated with at least one gentamicin dose during their ICU stay, we found low agreement despite low bias between eGFR_Gentamicin_ and eGFR_Creatinine_ respectively eGFR_CystatinC_. However, when we assessed the three eGFR methods as predictors for risk of RRT during the ICU stay and 30 day-mortality the three eGFR methods performed similarly with a trend towards higher c-index for eGFR_Gentamicin_.

The finding that gentamicin CL shows a low agreement compared to the other two endogenous eGFR methods is not surprising since it is well-known that neither eGFR_Creatinine_ nor eGFR_CystatinC_ is accurate in critically ill ICU patients^9–11^. One reason behind this is the pathophysiological changes in critically ill patients causing alterations in renal hemodynamics^9^. Modern interventions during ICU care can also affect both creatinine and cystatin C levels causing false estimation of GFR^9,17,18^.

Creatinine is produced in all muscle cells and its serum levels are influenced by gender, ethnicity, muscle mass, physical activity and diet among others^19,20^. Nutritional status and muscle wasting due to ICU care will affect the production of creatinine. The equations for eGFR are based on patient cohorts with low severity of illness without muscle wasting or nutrition deficit, contrary to ICU patients. We saw a small but consistent association in the bias, i.e. lack of agreement, between eGFR_Creatinine_ and eGFR_Gentamicin_ suggesting that muscle mass contributes to this. There are also other factors influencing creatinine in the critically ill that make this marker of kidney function less reliable in ICU situations, like AKI and dialysis^17,18^. Creatinine is also secreted in the renal tubules, which is another limitation since it may lead to false overestimation of CL compared to mGFR and making this biomarker an unreliable predictor of renal drug elimination and dosing of β-lactam antibiotics in the ICU^21,22^.

Cystatin C is generated in all nucleated cells and is less affected by muscle mass than creatinine but has been shown to be affected by levels of thyroid hormones, corticosteroids and possibly by obesity, smoking and inflammation^10,17,18,23–26^. Despite many improvements compared to eGFR_Creatinine_, a systematic review showed that in critically ill, eGFR_CystatinC_ both underestimates and overestimates renal function compared to measured GFR (mGFR) in five studies on ICU patients^10^.

Gentamicin on the other hand was in some small cohort studies in the 1980s and 1990s suggested to be a potential exogenous marker for GFR in patients with infections that already receive treatment with this compound in the ICU. Koren et al. reported that GFR in 38 preterm infants in the ICU could be estimated by using gentamicin pharmacokinetics calculations based on serum concentrations of gentamicin with good results compared to measured creatinine CL^27^. Zarowitz et al. found that aminoglycoside CL in ten tobramycin or gentamicin-treated ICU patients was as good as measured inulin CL and 24-hour urinary creatinine CL to estimate GFR in critically ill patients^15^. Hickling et al. on the other hand found that aminoglycoside (AG) CL is predicted as poorly by renal AG CL as by creatinine CL in critically ill patients and proposed that their findings suggested a non-renal aminoglycoside CL^14^. Jones et al. reported that AG CL was a good estimate of creatinine CL in 100 tobramycin or gentamicin-treated ICU patients compared to seven other used estimates^13^. For some reason, possibly due to the development of equations using cystatin C, these reports regarding gentamicin as an exogenous marker of GFR fell into oblivion. Compared to these early studies our study also focuses on comparing eGFR_Gentamicin_ to eGFR_CystatinC_ besides eGFR_Creatinine_ and we also estimate eGFR_Gentamicin_ using a population PK model-based method and renal endpoints for the first time.

Decreased eGFR is associated with poor survival and acute RRT during ICU stay^28,29^. Previous studies of eGFR have also reported that cystatin C alone or together with creatinine-based equations can be used to predict the risk for long-term cardiovascular death^30–32^. Our finding with similar c-index for eGFR_Gentamicin_ respectively eGFR_CystatinC_ in predicting the risk of 30-day mortality and risk for RRT during ICU stay strengthens the hypothesis that gentamicin CL could be as good as the other two eGFR biomarkers to estimate kidney function in the ICU. The three eGFR methods were also comparable when it comes to predicting the overall risk of death during the follow-up period which further strengthens this conclusion.

To our knowledge, this is the first study that tries to compare eGFR_Gentamicin_, estimated from serum gentamicin concentrations using a PK model-based approach, in a large ICU cohort of 254 patients to the modern equations for eGFR_Creatinine_ and eGFR_CystatinC,_ the standard estimates of GFR in the ICU in many countries. We also assess the eGFR_Gentamicin_ method as a predictor of both mortality and RRT during ICU care for the first time i.e. not only comparing it to other eGFR methods but to clinical outcomes related to renal function. However, the study has some limitations. Firstly, we did not compare the eGFR_Gentamicin_ with the reference standard of mGFR, inulin. Secondly, gentamicin is a drug with nephrotoxic potential and although unlikely, it cannot be ruled out that the correlation between eGFR_Gentamicin_ and the need for RRT during ICU stay could be partly explained by this fact. Another limitation is that the use of gentamicin has declined during the last decade and the usefulness of developing a new exogenous GFR method based on gentamicin can be questioned. However, in certain patient groups, like in the neonatal ICU, patients in septic shock, and in countries where gentamicin is used together with β-lactams as empirical treatment in infections with unknown cause because of antimicrobial stewardship reasons, the use of eGFR_Gentamicin_ could be a good alternative. In clinical practice, this means that eGFR_Gentamicin_ in the mentioned groups of patients could be used e.g. to guide dose adjustments of other renally cleared drugs like the β-lactam during the first critical days of treatment.

Analysis of gentamicin concentrations is also well-established in almost all hospitals which makes the method widely available with short test turnaround times. When it comes to the patient cohort no clinical data on the type of infection or underlying conditions were collected but on the other hand, an ICU cohort of 254 patients is relatively large and the finding of our study is applicable to a general ICU population.

In recent years, the development of several user-friendly software for estimating PK parameters such as CL^33^, as well as predicting individual dosing regimens, opens new possibilities for the clinical use of gentamicin concentrations derived from ICU patients to be used for estimating their individual GFR from estimated gentamicin CL. This could be beneficial for the critically ill population when it comes to both improved dosing of other renally cleared drugs like the β-lactams, but might also be a helpful tool for the intensive care doctors to use to predict the risk of mortality and need for early RRT.

Our findings support exploring the use of gentamicin as an exogenous marker of renal function further. Preferably, by comparing the eGFR_Gentamicin_, derived using a population PK model approach, to mGFR by iohexol, or possibly inulin, the reference standard, in an ICU population, with severe infections as a first step. A possible future application could be to integrate a renal function estimate based on gentamicin CL in an easy-to-use model-informed precision dosing software used for β-lactams to guide dosing in ICU patients during the first critical days of treatment^34^.

## Conclusion

In Swedish ICU patients treated with gentamicin, the use of estimated gentamicin CL from measured gentamicin serum concentrations was found to be a potential exogenous marker of renal function that needs to be explored further. eGFR_Gentamicin_ was also found to be as good as eGFR_Creatinine_ and eGFR_CystatinC_ in predicting mortality and the need for early RRT in the ICU.

### Ethical approval

The study was approved by the regional ethics review board in Uppsala (Registration number 2016/157) and conducted in accordance with the Declaration of Helsinki and its subsequent revisions. Due to the retrospective nature of the study, the regional ethics review board in Uppsala waived the need of obtaining informed consent. The STROBE statement was followed for reporting.

### Measurement of creatinine, Cystatin C and gentamicin

Measurement of plasma creatinine, cystatin C and serum concentrations of gentamicin were performed at the accredited Department of Clinical Chemistry and Pharmacology at Uppsala University Hospital. Both creatinine and cystatin C were analysed on Architect ci8200 (Abbot Laboratories, Abbot Park, Ill, USA). Plasma creatinine was analysed with an enzymatic method calibrated by the manufacturer using the isotope dilution mass spectrometric method (IDMS). Plasma cystatin C was analysed with an assay from Gentian (Gentian, Moss, Norway) calibrated against the international calibrator ERM-DA471/IFCC.

Serum gentamicin concentrations were analysed with two different methods during the study period. Between 2009 and 2011 the analysis was made using fluorescence polarization immune assay (F-PIA) on TDx Flex from Abbott, and from September 2011 with chemiluminescence microparticle immunoassay (CMIA) method on Architect, Abbot. The two methods produced comparable results according to the validation performed by the accredited laboratory.

#### Estimation of GFR

Gentamicin CL was estimated for each individual using Bayesian estimation^34^, applying the first measured concentration (one sample, 95% of samples collected 6–12 h after dose) and a population pharmacokinetic model described by Hodiamont et al.^35^. This is a two-compartmental model based on prospective data from 59 critically ill patients treated with a mean gentamicin dose of 5.1 mg/kg (± 1.1, SD). The data consisted of 416 gentamicin concentrations from TDM sampling (peak and random time point 6–23 h after administration) and measurements from waste material. The model includes inter-individual variability for CL (75%) and central volume of distribution (27%) but no covariates^35^. The model was selected following an evaluation of goodness-of-fit plots and a simulation-based prediction corrected visual predictive check^36^. Estimated CL (mL/min) was converted to relative value (mL/min/1.73m^2^) by applying an equation for body surface area^37^, and used as eGFR_Gentamicin_. eGFR_Creatinine_ and eGFR_CystatinC_ (mL/min/1.73m^2^) were calculated from the LM-rev and the CAPA-equations, respectively^38,39^.

### Statistics

To detect a 10 ml/min bias with a mean eGFR of 60 ml/min and a standard deviation of 30 ml/min, with an alpha of 0.05 and a power of 0.9, 191 patients needed to be included in the study.

Missing data for height (47 patients) and weight (20 patients) were imputed with the median from all patients in the study. No other variables had missing data.

Data are presented as median (IQR) or as number of observations (%) unless otherwise stated. Pearson’s correlation coefficients and coefficients of determination were used to assess correlations. The agreement and bias between eGFR_Gentamicin_ and eGFR_Creatinine_, respectively eGFR_CystatinC_ were calculated and presented in Bland-Altman plots. Univariate logistic regression, presented as receiver operating characteristic curves (ROC-curves), was used to assess the association between the three eGFR methods and the risk of RRT during ICU stay as well as 30-day mortality. The association between the three eGFR methods and overall mortality during the follow-up period was analysed using Cox proportional hazard regression.

### Software

Statistica software, version 14.1 (Statsoft, Tulsa, OK, USA) was used for the statistics. Gentamicin CL was estimated using Bayesian estimation in the software NONMEM (version 7.4, Icon Development Solutions, Hanover, MD, USA)^40^, assisted by Pearl-Speaks-NONMEM^41^. R version 3.5 (R Foundation for Statistical Computing, Vienna, Austria) was used for data management, with the xpose4 package^41^, for population PK model evaluation.

## Electronic supplementary material

Below is the link to the electronic supplementary material.

Supplementary Material 1

## Data Availability

Anonymised datasets generated during the study are available from the corresponding author on reasonable request.

## Abbreviations

AKI: Acute kidney injury
ARC: Augmented renal clearance
BSA: Body surface area
CL: Clearance
eGFR: Estimated glomerular filtration rate
eGFR_Creatinine_: Estimated glomerular filtration rate based on creatinine (LM-rev equation)
eGFR_CystatinC_: Estimated glomerular filtration rate based on cystatin C (CAPA-equation)
eGFR_Gentamicin_: Estimated glomerular filtration rate based on gentamicin clearance
ICU: Intensive care unit
mGFR: Measured glomerular filtration rate
RRT: Renal replacement therapy
