# Supplementary material for: Utilizing gentamicin concentrations to estimate glomerular filtration rate in intensive care unit patients
Source: Sci Rep. 2025 May 18;15:17262. doi: 10.1038/s41598-025-01256-z (PMC12086182; doi:10.1038/s41598-025-01256-z)
Supplement: Supplementary file 1 — Supplementary Material 1 [file 41598_2025_1256_MOESM1_ESM.docx]

## **Supplementary file for the manuscript;**

**Utilizing gentamicin concentrations to estimate glomerular filtration rate in intensive care patients**

Authors: Anna-Karin Smekal, Maria Swartling, Elisabet I. Nielsen, Mia Furebring, Anders O. Larsson, Miklos Lipcsey

Table S1. Comparison of eGFR (eGFR_Creatinine_, eGFR_CystatinC_, eGFR_Gentamicin_) in patients with height and weight data available and those with missing data.

|  | **Height data available** | **Height data missing** |
| --- | --- | --- |
| eGFR_Creatinine_^1^, ml/min/1.73m^2^ BSA | 55 (35 - 83) | 57 (25 - 78) |
| eGFR_CystatinC_^2^, ml/min/1.73m^2^ BSA | 45 (28 - 70) | 53 (28 - 76) |
| eGFR_Gentamicin_^3^, ml/min/1.73m^2^ BSA | 48 (31 - 76) | 52 (27 - 67) |
|  |  |  |
|  | **Weight data available** | **Weight data missing** |
| eGFR_Creatinine_^1^, ml/min/1.73m^2^ BSA | 55 (34 - 82) | 55 (24 - 85) |
| eGFR_CystatinC_^2^, ml/min/1.73m^2^ BSA | 45 (28 - 72) | 50 (28 - 69) |
| eGFR_Gentamicin_^3^, ml/min/1.73m^2^ BSA | 49 (31 - 73) | 46 (31 - 81) |

BSA, body surface area; eGFR, estimated glomerular filtration rate; ICU, intensive care unit.

^1^LM-Rev-equation ^2^CAPA-equation ^3^Gentamicin-clearance using population PK-model by Hodiamont

Data are presented as median (IQR) or number (percentages).

Table S2. Comparison of eGFR (eGFR_Creatinine_, eGFR_CystatinC_, eGFR_Gentamicin_) in female and male patients .

|  | **Female patients** | **Male patients** |
| --- | --- | --- |
| eGFR_Creatinine_^1^, ml/min/1.73m^2^ BSA | 58 (32-88) | 52 (34-78) |
| eGFR_CystatinC_^2^, ml/min/1.73m^2^ BSA | 53 (28-79) | 43 (28-65) |
| eGFR_Gentamicin_^3^, ml/min/1.73m^2^  BSA | 48 (28-72) | 50 (31-76) |

BSA, body surface area; eGFR, estimated glomerular filtration rate; ICU, intensive care unit.

^1^LM-Rev-equation ^2^CAPA-equation ^3^Gentamicin-clearance using population PK-model by Hodiamont

Data are presented as median (IQR) or number (percentages).
